# The role of β-adrenergic stimulation in QT interval adaptation to heart rate during stress test

**DOI:** 10.1371/journal.pone.0280901

**Published:** 2023-01-26

**Authors:** Cristina Pérez, Rubén Cebollada, Konstantinos A. Mountris, Juan Pablo Martínez, Pablo Laguna, Esther Pueyo

**Affiliations:** 1 BSICoS Group, I3A, University of Zaragoza, Zaragoza, Spain; 2 IIS Aragón, Zaragoza, Spain; 3 CIBER de Bioingeniería, Biomateriales, y Nanomedicina, Instituto de Salud Carlos III, Zaragoza, Spain; Universidade Federal de Sao Joao del-Rei, BRAZIL

## Abstract

The adaptation lag of the QT interval after heart rate (HR) has been proposed as an arrhythmic risk marker. Most studies have quantified the QT adaptation lag in response to abrupt, step-like changes in HR induced by atrial pacing, in response to tilt test or during ambulatory recordings. Recent studies have introduced novel methods to quantify the QT adaptation lag to gradual, ramp-like HR changes in stress tests by evaluating the differences between the measured QT series and an estimated, memoryless QT series obtained from the instantaneous HR. These studies have observed the QT adaptation lag to progressively reduce when approaching the stress peak, with the underlying mechanisms being still unclear. This study analyzes the contribution of *β*-adrenergic stimulation to QT interval rate adaptation in response to gradual, ramp-like HR changes. We first quantify the QT adaptation lag in Coronary Artery Disease (CAD) patients undergoing stress test. To uncover the involved mechanisms, we use biophysically detailed computational models coupling descriptions of human ventricular electrophysiology and *β*-adrenergic signaling, from which we simulate ventricular action potentials and ECG signals. We characterize the adaptation of the simulated QT interval in response to the HR time series measured from each of the analyzed CAD patients. We show that, when the simulated ventricular tissue is subjected to a time-varying *β*-adrenergic stimulation pattern, with higher stimulation levels close to the stress peak, the simulated QT interval presents adaptation lags during exercise that are more similar to those measured from the patients than when subjected to constant *β*-adrenergic stimulation. During stress test recovery, constant and time-varying *β*-adrenergic stimulation patterns render similar adaptation lags, which are generally shorter than during exercise, in agreement with results from the patients. In conclusion, our findings support the role of time-varying *β*-adrenergic stimulation in contributing to QT interval adaptation to gradually increasing HR changes as those seen during the exercise phase of a stress test.

## 1. Introduction

Ventricular arrhythmias and sudden cardiac death are major causes of mortality and disability in patients with Coronary Artery Disease (CAD) or myocardial infarction (MI). Due to the limitations of commonly employed clinical risk markers, a large number of indices calculated from resting or ambulatory electrocardiograms (ECGs) have been proposed over the years to stratify CAD and MI patients for arrhythmic risk [1–5]. In particular, indices have been developed to quantify characteristics of the QT interval or the T wave of the ECG that could provide relevant information on enhanced temporal and spatial dispersion of ventricular repolarization favoring arrhythmia development [6–13]. One of these indices measures the adaptation lag of the QT interval in response to heart rate (HR) changes, a phenomenon termed QT hysteresis [14–16]. The potential of QT hysteresis for arrhythmic risk stratification has been established in different clinical populations, particularly in post-myocardial infarction patients [17–19].

The rate adaptation of the QT interval has been most usually evaluated in response to abrupt changes in HR, either in Holter recordings, in response to a tilt test or following atrial pacing [14, 17, 20–22]. Nevertheless, abrupt, step-like HR changes are not always easily observed or even induced. Recent studies have proposed alternative methods to evaluate QT rate adaptation in response to more gradual, ramp-like increases in HR as those observed during stress tests [23, 24]. These studies have quantified the QT adaptation lag based on the differences between the measured QT interval time series and an estimated, memoryless QT time series computed as the output of a regression model when the input is the RR interval time series. It should be noted that if the relationship between the QT and RR interval time series were represented by a first-order system, as suggested from previously reported results [14, 17, 21], the QT response to a step-like or a ramp-like HR change could be characterized by the same time constant in the two cases [25]. Measuring the adaptation to ramp-like HR changes has the advantage that these types of change are easier to observe, e.g. in a stress test, than step-like HR changes.

In addition to HR, other physiological factors influence the QT interval, with the Autonomic Nervous System (ANS) being an important one [26–28]. The ANS modulates ventricular repolarization both directly by its action on the ventricular myocardium and indirectly by HR-related effects [29–33]. Consequently, the ANS can exert an impact not only on the duration of the QT interval but also on its adaptation to HR changes, thus contributing to modulate QT hysteresis. Indeed, studies evaluating QT hysteresis from stress test recordings have described a progressive reduction in the QT interval adaptation lag when approaching the stress test peak [23, 24]. The mechanisms underlying such a QT lag reduction are yet unclear. *In silico* studies have investigated the dynamics of cellular repolarization duration in response to *β*-adrenergic stimulation [34, 35] and have shown that the time lag of the action potential (AP) duration (APD) in response to *β*-adrenergic stimulation becomes reduced for higher pre-stimulation levels of *β*-adrenoceptors [36]. Based on these studies, we hypothesized that the enhanced sympathetic activity when getting closer to the stress peak, and thus increasingly higher *β*-adrenergic stimulation, could play a role in the observed QT lag reduction at the end of the exercise. More generally, we hypothesized that a time-varying *β*-adrenergic stimulation pattern along a stress test could contribute to explain the profiles of QT rate adaptation in patients.

We investigated the role of *β*-adrenergic stimulation in QT interval rate adaptation. We first quantified the QT adaptation lag in a set of CAD patients undergoing a stress test. Next, we used biophysically detailed cell models coupling mathematical formulations of human ventricular electrophysiology and *β*-adrenergic signaling and we generated pseudo-electrocardiogram (pECG) signals from modeled transmural ventricular tissue fibers. We conducted simulations to characterize QT rate adaptation in response to each of the HR time series measured from CAD patients during stress test. We evaluated the extent to which time-varying *β*-adrenergic stimulation patterns, in contrast to constant *β*-adrenergic stimulation, contribute to better explain the QT adaptation delays measured from the patients.

## 2. Materials and methods

### 2.1 ECG recordings

ECG recordings from CAD patients performing a stress test in a bicycle ergometer were collected at Tampere University Hospital, Finland [37]. 12 ECG recordings were randomly selected, four from each of the groups clustered according to the degree of CAD: low-CAD, defined by less than 50% of luminal narrowing of the diameter of at least one major epicardial coronary artery or main branches; mild-CAD, defined between 50 and 75% of narrowing; and high-CAD, defined by more than 75% of narrowing. ECG was recorded at a sampling frequency *F*_*s*_ = 500 Hz. The initial workload varied from 20 W to 30 W, depending on the patient, and the load was increased stepwise by 10–20 W every minute [37]. The study protocol was approved by the Ethical Committee of the Hospital District of Pirkanmaa, Finland, and all patients gave informed consent prior to the interview and measurements as stipulated in the Declaration of Helsinki.

### 2.2 ECG processing to compute RR and QT interval series

ECG signals were filtered to remove baseline wander, high-frequency noise and artifacts. A single-lead wavelet-based algorithm [38] was applied to delineate the 8 available independent standard leads and, subsequently, a multi-lead strategy was used to define a unique mark for the R wave and for the QRS onset of each beat. From the computed delineation marks, the time series of the RR interval (between consecutive R wave peaks) were calculated for each recording. To compute the T wave end of each beat, a spatially-transformed lead derived from Periodic Component Analysis (*π*CA) [39] focused on windows around the T waves was calculated. The same single-lead wavelet-based algorithm described above was applied onto the calculated lead to obtain the T wave end delineation marks. From the identified QRS onset and T wave end marks, the time series of the QT interval (from the QRS onset to the T wave end) was computed for each recording.

Outlier values in the RR interval time series (QT interval time series, respectively) were identified as those deviating by more than ±10% (±5%, respectively) from the running median of each series computed over 40 beats. The identified outliers were replaced with the running median value [23]. Next, the unevenly sampled RR and QT interval time series were interpolated to 4 Hz to obtain uniformly sampled *d*_RR_(*n*) and *d*_QT_(*n*) intervals series, respectively, measured in seconds.

### 2.3 *In silico* ventricular cell models

To investigate the mechanisms underlying QT interval adaptation to HR changes during stress test, we conducted an *in silico* modeling and simulation study covering multiple scales from cell to ECG. We evaluated the APD in simulated cells and the QT interval in simulated pseudo-electrocardiograms, pECGs, in response to changes in HR as those measured from the patients.

At the cellular level, we represented the electrophysiology of human ventricular cardiomyocytes by using the O’Hara et al. AP model [40]. In particular, we used the three models proposed by O’Hara and coworkers to describe the electrical activity of subendocardial, midmyocardial and subepicardial cells across the ventricular wall. These models included descriptions of the main ionic currents and fluxes involved in the generation of the AP in the three cell types. The relationship between the ionic currents and fluxes and the transmembrane potential were obtained by solving the equation:

$$\begin{array}{cc} C_{m}\frac{dV(t)}{dt}\; & +\;\sum_{s}g_{s}\left(V\left(t\right)-E_{s}\right)\;+\;\sum_{b}I_{b}\left(t\right)\;+\\ & +\;\sum_{i}I_{i}\left(t\right)\;+\;I_{st}\left(t\right)=0 \end{array}$$ (1)

where *C*_*m*_ is the membrane capacitance per unit of area, *V* is the transmembrane potential, *g*_*s*_ is the conductance of the ionic current for ion species *s*, *E*_*s*_ is the equilibrium potential of ion *s*, *I*_*b*_ is the current through pump *b*, *I*_*i*_ is the current through exchanger *i* and *I*_*st*_ is the stimulus current. The formulation of the fast sodium current in the O’Hara et al. models was replaced with the formulation by ten Tusscher et al. following the comment by O’Hara and coworkers [40, 41] (comment on article from 05 Oct 2012).

The described electrophysiological models for the three ventricular cell types were coupled to the Gong et al. model of *β*-adrenergic receptor signaling [42]. The Gong et al. model represents an adaptation from the Heijman et al. model originally developed for the canine myocyte [43]. The model describes the AP response to different concentrations of the *β*-adrenergic agonist isoproterenol (Iso) by computing each ionic current or flux as a weighted average of the phosphorylated and nonphosphorylated fractions of each cellular substrate. Departing from the Heijman et al. model, the Iso-induced changes in individual substrates like the L-type Ca^2+^ channels, slow delayed rectifier K^+^ channels and ryanodine receptors were calibrated based on experimental data [42]. Further adjustments in the model were performed by Gong et al. to reproduce whole-cell healthy human ventricular AP data at maximal *β*-adrenergic stimulation under a range of pacing frequencies.

To evaluate the cellular APD response to changes in HR as those measured from the patients during stress test, we paced the cells according to the RR intervals calculated from the analyzed ECG recordings. For each simulated beat, the APD in response to each given RR interval was computed as the AP duration at 90% repolarization. The APD time series sampled at 4 Hz was denoted as *d*_APD_(*n*).

### 2.4 *In silico* ventricular tissue models and simulation of ECG signals

Based on the human ventricular cell models described in section 2.3, we next evaluated the response to HR changes of a human ventricular tissue. We considered a ventricular transmural tissue fiber of length *L* = 1.5 cm composed of subendocardial, midmyocardial and subepicardial cells. Electrical propagation in the tissue was represented by the monodomain model, which is a simplified version of the bidomain model [44, 45]. The monodomain model is described by a reaction-diffusion partial differential equation (PDE) for the transmembrane potential, with the extracellular potential being calculated from another PDE once the transmembrane potential has been solved. Propagation of the transmembrane potential in the tissue was described by the following equation:

$$\begin{array}{c} \frac{\partial V(x,t)}{\partial t}=\frac{-I_{ion}\left(V\left(x,t\right)\right)}{C_{m}}+D\frac{\partial^{2}V\left(x,t\right)}{\partial x^{2}} \end{array}$$ (2)

where $\frac{\partial V(x,t)}{\partial t}$ is the partial time derivative of the transmembrane potential at a point *x* in the tissue fiber and a time *t*, $\frac{\partial^{2}V\left(x,t\right)}{\partial x^{2}}$ is the second partial space derivative of the transmembrane potential at a point *x* in the tissue fiber and a time *t*, *I*_*ion*_ is the total ionic current (calculated as the sum of all the terms except for the first one in Eq (1), with the stimulus current corresponding to the one applied onto the fiber) and *D* is the diffusion coefficient. Zero-flux Neumann boundary conditions were imposed.

Cellular heterogeneities were defined according to the following transmural distribution across the tissue fiber: 45% of subendocardial cells, 25% of midmyocardial cells and 30% of subepicardial cells, in line with previous studies based on experimentally reported values [46–48].

The value of the diffusion coefficient *D* was set so as to have a conduction velocity value close to 70 cm/s in the fiber, which is within the physiological ranges reported in experimental studies [49, 50].

The reaction-diffusion PDE of the monodomain model was solved by using the operator splitting method [51] to decouple the reaction term, which describes the generation of the cellular AP, and the diffusion term, which describes the AP propagation in the tissue. The decoupled PDE was solved by the Finite Element Method (FEM) using the ELECTRA solver [52, 53] with a spatial resolution of 0.015 cm. Numerical time integration was performed using a dual adaptive explicit time integration algorithm [54].

**Fig 1:**
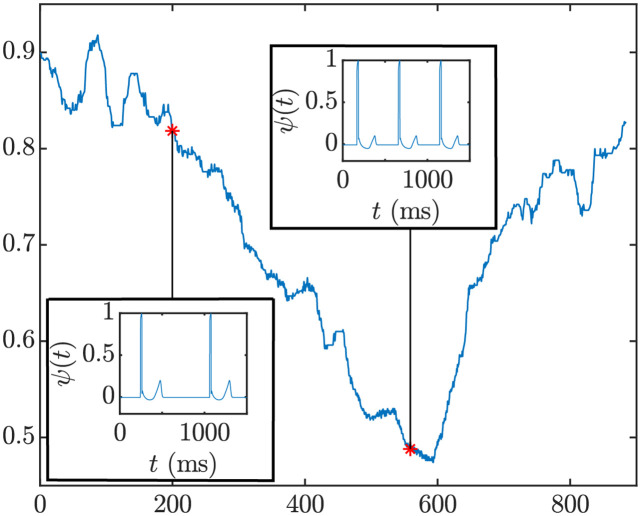
Main panel: *d*_RR_(*n*) time series, in seconds, from the analyzed ECG recording of a CAD patient. Insets: normalized pECG signals, *ψ*(*t*), computed from the simulated transmembrane potentials of the tissue fiber at two time instants.

Taking the simulated transmembrane potentials from the cardiac tissue fiber, we computed a pECG signal representing the extracellular potential recorded by an electrode placed 2 cm away from the subepicardial end of the fiber in the direction of the fiber axis, as in previous studies investigating ventricular repolarization from simulated transmural one-dimensional tissues [55]. The pECG signal, *ψ*(*t*), was calculated at each time instant *t* using the following equation and was subsequently normalized to have unit amplitude:

$$\begin{array}{c} \psi \left(x^{\prime },t\right)=-\int_{x=0}^{x=L}\sigma \;\nabla_{x}V\left(x,t\right)\cdot \nabla_{x}(\frac{1}{r(x,x^{\prime })})\;dx, \end{array}$$ (3)

where *σ* is the diffusion coefficient of the electrical medium surrounding the tissue, *L* is the fiber length and *r*(*x*, *x*′) is the distance between a point x within the fiber and the recording electrode located at a point x’ outside the fiber but in the fiber axis direction. Fig 1 shows the pECG waveforms (restricted to the ventricular activity) calculated at two time instants along the stress test after having paced the tissue fiber following the *d*_RR_(*n*) time series of a CAD patient.

The QT interval was measured on the pECG by using the same wavelet-based delineation method applied to the ECG signals acquired from the patients. The simulated QT time series at 4 Hz was denoted as *d*_QT_(*n*).

### 2.5 Estimation of the memoryless QT/RR relationship

To model the instantaneous, memoryless dependence of the QT interval (APD, respectively) on the RR interval, linear and hyperbolic regression models were used, as these have been reported to represent the most common patterns of the QT-to-RR relationship [17]:

$$\begin{array}{cccc} \text{Linear} & \;\;\text{(Lin)} & d_{\text{QT}}^{i}\left(n\right) & =\beta +\alpha \;d_{\text{RR}}\left(n\right)\\ \text{Hyperbolic} & \;\;\text{(Hyp)} & d_{\text{QT}}^{i}\left(n\right) & =\beta +\frac{\alpha }{d_{\text{RR}}\left(n\right)} \end{array}$$ (4)

where the QT and RR intervals are expressed in seconds and the notation $d_{\text{QT}}^{i}\left(n\right)$ ($d_{\text{APD}}^{i}\left(n\right)$, respectively) is used to emphasize that the output of the regression model is a time series of QT intervals or APD values that depends on the instantaneous RR and not on a previous history of RR intervals.

To estimate the values of the parameters *α* and *β* in the regression models, the QT (APD, respectively) and RR interval time series of each patient were fitted in time windows where the RR interval was considered to be stationary and, thus, the QT interval (APD) was considered to be mostly dependent on the immediately previous RR interval rather than on other past RR intervals. Using the estimated values of *α* and *β* in the regression formulas, the time series $d_{\text{QT}}^{i}\left(n\right)$ ($d_{\text{APD}}^{i}\left(n\right)$, respectively) was calculated for the whole stress test using Eq (4).

The three time windows of RR stationarity for regression parameter estimation were taken as follows: *W*_1_, at the beginning of the stress test (40 s); *W*_2_, around the stress peak (20 s); and *W*_3_, at the end of the test (40 s) [23]. Data in window *W*_2_ was taken duplicated so as to have as much data in each stationary window with low HR as in the stationary window with high HR. In the following, when using *W*_2_, we will refer to the duplicated data. The window *W*_2_ around the stress peak was included to guarantee a large enough range of RR intervals to fit the QT-to-RR (APD-to-RR) regression models. However, since it may be questionable to assume RR stationarity in that time window, in the next section we describe two strategies used to mitigate this effect and more robustly determine the instantaneous QT-to-RR (APD-to-RR) dependence. The “unmodified” strategy (U) used the values of the QT (APD, respectively) time series in the window *W*_2_ without any modification to fit the regression models. The “modified” strategy (M) redefined the values of the QT (APD, respectively) time series in the window *W*_2_ to account for the ones that would have been attained if RR values had remained stationary at the exercise peak for some additional time.

#### 2.5.1 Estimation from unmodified QT series

In the “unmodified” strategy (U), a symmetric window centered on the stress peak, i.e. with the same duration at both sides of the peak, was chosen as *W*_2_. In this way, the decreasing values of RR and QT (APD) in the exercise phase were considered to be somehow compensated by the increasing values of RR in the recovery phase. This was hypothesized to provide an overall QT-to-RR (APD-to-RR) relationship equivalent to the one that would be obtained if stationarity had being reached. The patient-specific regression parameter values calculated by using this strategy were denoted by ${\hat{\alpha }}_{u}$ and ${\hat{\beta }}_{u}$, where the subscript *u* denotes estimation from unmodified data.

#### 2.5.2 Estimation from modified QT series

In the “modified” strategy (M), an initial value of the time delay between the measured QT series *d*_QT_(*n*) and the estimated memoryless QT series $d_{\text{QT}}^{i}\left(n\right)$ was calculated in the exercise phase of the stress test based on the above definition of *W*_2_ and the methodology described in section 2.6. The values of *d*_QT_(*n*) in the *W*_2_ window were modified according to:

$$\begin{array}{c} d_{{\text{QT}}_{m}}\left(n\right)=d_{\text{QT}}\left(n\right)-\Delta \text{QT} \end{array}$$ (5)

where *Δ*QT = *s*_*e*,*u*_ × *τ*_*e*,*u*_, being *s*_*e*,*u*_ the absolute value of the slope of the QT series in the exercise peak and *τ*_*e*,*u*_ the initially estimated value of the time delay. Thus, this strategy M considered the estimates of the QT values that would have been attained provided the RR had reached stationarity if the exercise phase had remained at the peak RR for an extra time *τ*_*e*,*u*_. Using the modified definition of QT in *W*_2_, patient-specific regression parameter values were calculated and denoted by ${\hat{\alpha }}_{m}$ and ${\hat{\beta }}_{m}$, where the subscript *m* denotes estimation from modified data.

For each patient, the “unmodified” U and “modified” M strategies were applied using both the linear and hyperbolic models. The model that generated the lowest residual *ε*_rms_ between *d*_QT_(*n*) and $d_{\text{QT}}^{i}\left(n\right)$, globally in the three stationary windows *W*_*j*_, *j* = 1, 2, 3, was selected:

$$\begin{array}{c} \varepsilon_{\text{rms}}=\sqrt{\frac{1}{3\times 40\times 4}\sum_{n\in \{W_{j}\}}{\left(d_{\text{QT}}^{i}\left(n\right)-d_{\text{QT}}\left(n\right)\right)}^{2}}, \end{array}$$ (6)

where 3 × 40 × 4 is the number of samples of the difference vector $d_{\text{QT}}^{i}\left(n\right)-d_{\text{QT}}\left(n\right)$ in the 3 windows of analysis, as each of the windows has a duration of 40 s and the $d_{\text{QT}}^{i}\left(n\right)$ and *d*_QT_(*n*) time series are interpolated to 4 Hz. Note that, in Eq (6), *d*_QT_(*n*) was replaced with $d_{{\text{QT}}_{m}}\left(n\right)$ in *W*_2_ when using the M strategy.

**Fig 2:**
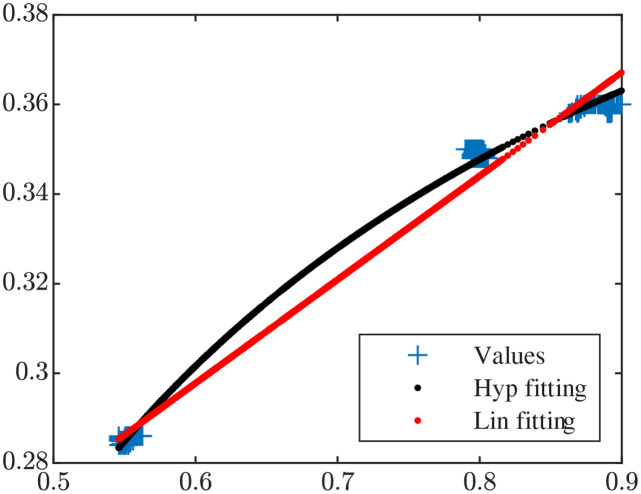
Fitting of linear (red) and hyperbolic (black) regression models to a patient’s QT and RR data (“highCAD-4” in Table 1). The data clusters correspond to the different windows *W*_*i*_, *i* ∈ 1, 2, 3. The residual *ε*_rms_ took values of 4.27 and 1.75 ms for the linear and hyperbolic fittings, respectively.

An example of how the two regression models were fitted to a patient’s QT and RR data in the windows *W*_1_, *W*_2_ and *W*_3_ using the U strategy is presented in Fig 2. As can be seen from the figure, the hyperbolic model led to the best fitting, which was corroborated by the quantitative results shown in Table 1.

**Table 1:** Left column: codes used to identify the analyzed patients according to the risk group and the patient order number # within the group. Middle column: QT adaptation delay values measured in the exercise and recovery phases of the stress test for each patient, denoted by *τ*_*e*,*p*_ and *τ*_*r*,*p*_ with *p* indicating estimated from patients’ data. Right column: mean square error *ε*_*rms*_ for the linear and hyperbolic regression models calculated using the U strategy.

	QT lags (s)		*ε*_*rms*_ (ms)	
	*τ*_*e*, *p*_	*τ*_*r*, *p*_	Lin	Hyp
**low-CAD1**	16.9	91.3	12.0	2.7
**low-CAD2**	100.8	44.4	7.3	7.4
**low-CAD3**	27.2	44.0	13.4	4.9
**low-CAD4**	59.5	2.7	8.1	7.5
**mild-CAD1**	38.2	42.9	12.5	7.4
**mild-CAD2**	27.9	49.6	3.6	2.2
**mild-CAD3**	4.4	21.6	2.8	2.0
**mild-CAD4**	57.3	33.2	4.3	3.8
**high-CAD1**	54.9	24.2	5.2	6.7
**high-CAD2**	74.6	38.5	1.8	3.4
**high-CAD3**	73.2	25.2	5.2	6.7
**high-CAD4**	22.3	59.4	4.2	1.7

Once the optimal regression model was selected for each patient and strategy, the time series $d_{\text{QT}}^{i}\left(n\right)$ was calculated along the complete stress test according to the expression of the model in Eq (4).

The procedure described above to compute the memoryless time series $d_{\text{QT}}^{i}\left(n\right)$ for the analyzed patients was also applied to compute the memoryless time series $d_{\text{QT}}^{i}\left(n\right)$ for simulated pECGs and memoryless time series $d_{\text{APD}}^{i}\left(n\right)$ for simulated cells.

### 2.6 Quantification of repolarization adaptation time

To estimate the QT (or APD) adaptation lag behind HR changes, a measure of the time delay between the memoryless $d_{\text{QT}}^{i}\left(n\right)$ and the measured *d*_QT_(*n*) time series was computed over time intervals of the stress test where HR presented linear changes, separately in the exercise and recovery phases. The optimal delay value τ* was searched for by using a Least Square Error (LSE) criterion to minimize the difference between *d*_QT_(*n*) and $d_{\text{QT}}^{i}\left(n-\tau \right)$ separately in the exercise and recovery phases of the stress test, obtaining delays denoted as *τ*_*e*_ and *τ*_*r*_, respectively. The minimization was performed over time intervals with the following limits in each of the two phases: exercise onset, *n*_*e*, *o*_; exercise end, *n*_*e*, *e*_; recovery onset, *n*_*r*, *o*_; and recovery end, *n*_*r*, *e*_.

The time point of exercise onset *n*_*e*, *o*_ was defined as the point leading to the minimum LSE between $d_{\text{QT}}^{i}\left(n\right)$ and a function defined during the exercise phase by a constant line (plateau) up to *n*_*e*, *o*_ followed by a line with negative slope after *n*_*e*, *o*_. Analogously, *n*_*r*, *e*_ was defined by minimizing the LSE between $d_{\text{QT}}^{i}\left(n\right)$ and a function defined in the recovery phase by a line with positive slope up to *n*_*r*, *e*_ followed by a plateau after it. Specifically, the search for *n*_*e*, *o*_ (analogously, *n*_*r*, *e*_) resulted from minimizing the following cost function [56]:

$$\begin{array}{c} n_{e,o}=\text{arg}\min_{k}J\left(k\right), \end{array}$$ (7)

where

$$\begin{array}{c} J\left(k\right)\;=\;\sum_{n=M_{1}}^{k-1}{\left(d_{\text{QT}}^{i}\left(n\right)\;-\;f_{b}\left(n\right)\right)}^{2}\;+\;\sum_{n=k}^{M_{2}}{\left(d_{\text{QT}}^{i}\left(n\right)\;-\;f_{a}\left(n\right)\right)}^{2}. \end{array}$$ (8)

The functions *f*_*b*_(*n*) = *a*_*b*_ + *b*_*b*_*n* and *f*_*a*_(*n*) = *a*_*a*_ + *b*_*a*_*n* were the linearly fitted models of the $d_{\text{QT}}^{i}\left(n\right)$ time series before and after the candidate sample point *k*, respectively, being *M*_1_ = 1 and *M*_2_ = *n*_*p*_ − 800, with *n*_*p*_ the time point corresponding to the stress peak. It should be noted that 800 samples correspond to 200 s, since the time series were interpolated to 4 Hz. Similarly, *n*_*r*, *e*_ was searched for by an analogous minimization procedure, but setting *M*_1_ = *n*_*p*_ + 320 and *M*_2_ as the last time point of the $d_{\text{QT}}^{i}\left(n\right)$ time series.

The time point corresponding to the end of the exercise ramp, *n*_*e*, *e*_, was defined as the time point for which $d_{\text{QT}}^{i}\left(n\right)$ shortened with respect to *n*_*e*, *o*_ by a percentage 100*γ*_*e*_ % of the total reduction reached at the stress peak:

$$\begin{array}{c} n_{e,e}\;\;=\;\;\text{arg}\min_{n}\;(|d_{\text{QT}}^{i}\left(n\right)\;\;-\;\;d_{\text{QT}}^{i}\left(n_{e,o}\right)\;+\;\gamma_{e}(d_{\text{QT}}^{i}\left(n_{e,o}\right)\;\;-\;\;d_{\text{QT}}^{i}\left(n_{p}\right))|). \end{array}$$ (9)

where the minimization was performed over the time points in the exercise phase. Similarly, the time point corresponding to the onset of the recovery ramp, *n*_*r*, *o*_, was identified as the time point for which $d_{\text{QT}}^{i}\left(n\right)$ increased with respect to the stress peak by a percentage 100*γ*_*r*_ % of the total increase reached at *n*_*r*, *e*_:

$$\begin{array}{c} \;\;n_{r,o}\;\;=\;\;\text{arg}\min_{n}\;(|d_{\text{QT}}^{i}\left(n\right)\;\;-\;\;d_{\text{QT}}^{i}\left(n_{r,e}\right)\;+\;\left(1\;\;-\;\;\gamma_{r}\right)\;(d_{\text{QT}}^{i}\left(n_{r,e}\right)\;\;-\;\;d_{\text{QT}}^{i}\left(n_{p}\right))|).\;\; \end{array}$$ (10)

where the minimization was performed over the time points in the recovery phase.

The threshold values *γ*_*e*_ and *γ*_*r*_ were chosen as 0.55 [23].

### 2.7 Simulated *β*-adrenergic stimulation patterns

The simulated APD responses to HR changes were analyzed for four different patterns of *β*-adrenergic stimulation. The first pattern (Iso-c) was defined by a constant level of *β*-adrenergic stimulation that corresponded to a fixed Iso concentration of 0.005 *μ*M, considered as a baseline level.

**Fig 3:**
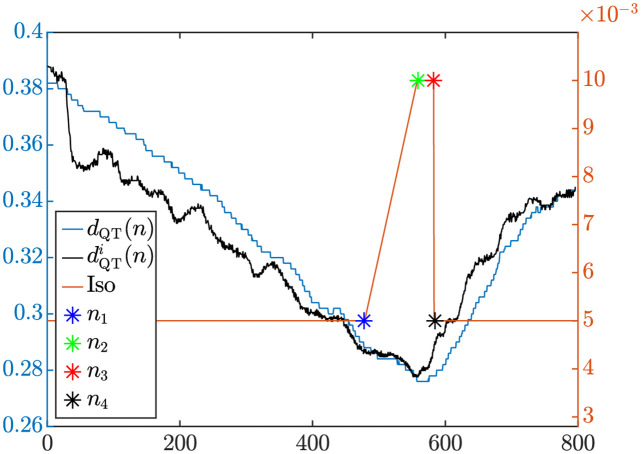
Time-varying pattern of Iso concentration during exercise and recovery, with time points *n*_1_, *n*_2_, *n*_3_ and *n*_4_ determined from the $d_{\text{QT}}^{i}\left(n\right)$ and *d*_QT_(*n*) time series of an analyzed ECG recording from a CAD patient.

The second pattern (Iso-tv) was defined by a time-varying level of *β*-adrenergic stimulation that comprised an increase in the stimulation when approaching the stress peak during the exercise phase and a reduction in the stimulation shortly after the start of the recovery phase. More precisely, this pattern departed from the baseline Iso concentration of 0.005 *μ*M and, at a time point *n*_1_ during exercise, the Iso concentration started to linearly increase until reaching a concentration of 0.01 *μ*M at the stress peak (*n*_2_ = *n*_*p*_). This concentration value was kept constant at the start of the recovery until time point *n*_3_. From *n*_3_ point onwards, Iso linearly dropped until time point *n*_4_, when it reached the baseline concentration. This is illustrated in Fig 3 for points *n*_1_, *n*_2_, *n*_3_ and *n*_4_ derived from the ECG data of a CAD patient.

To determine the four time points *n*_1_, *n*_2_, *n*_3_ and *n*_4_ defining the time-varying *β*-adrenergic stimulation pattern, we departed from the *d*_QT_(*n*) and *d*_RR_(*n*) time series of each patient and we computed the $d_{\text{QT}}^{i}\left(n\right)$ time series. The four time points were obtained according to the following criteria, which were based on the examination of the QT and RR time series of all analyzed patients:

- *n*_1_: time point closest to the stress peak, and prior to it, for which the slopes of *d*_QT_(*n*) and $d_{\text{QT}}^{i}\left(n\right)$ are equal.
- *n*_2_: time point corresponding to the stress peak *n*_*p*_.
- *n*_3_: first time point during recovery for which the difference between *d*_QT_(*n*) and $d_{\text{QT}}^{i}\left(n\right)$ slopes presents a local maximum.
- *n*_4_: *n*_3_ + 1, representing an abrupt reduction in the Iso concentration soon after the start of the recovery phase.

Two other patterns of *β*-adrenergic stimulation were investigated departing from the Iso-tv pattern. For the linearly increasing pattern (Iso-li), the Iso concentration was initially equal to 0.005 *μ*M and varied linearly from the exercise onset to the exercise peak, where it reached a value of 0.01 *μ*M. The rest of the Iso curve was the same as for Iso-tv. For the abruptly changing pattern (Iso-ab), the Iso concentration rose abruptly at the exercise onset from 0.005 *μ*M to 0.01 *μ*M and remained at this value until the exercise peak. The rest of the Iso curve was as for Iso-tv.

The four *β*-adrenergic stimulation patterns are illustrated in S1 Fig in S1 File. Based on the results at the cellular level, the simulated QT responses to HR changes were analyzed only for the Iso-c and Iso-tv patterns.

## 3 Results

### 3.1 QT adaptation to HR changes in patients

Table 1, middle column, shows the values of the QT adaptation delay, calculated using the strategy U, in the exercise and recovery phases of stress test recordings from CAD patients. The right column shows the root mean square error *ε*_*rms*_ calculated for each of the two tested regression models, from which the optimal model was chosen individually for each patient.

### 3.2 APD adaptation to HR changes in simulated cells

**Fig 4:**
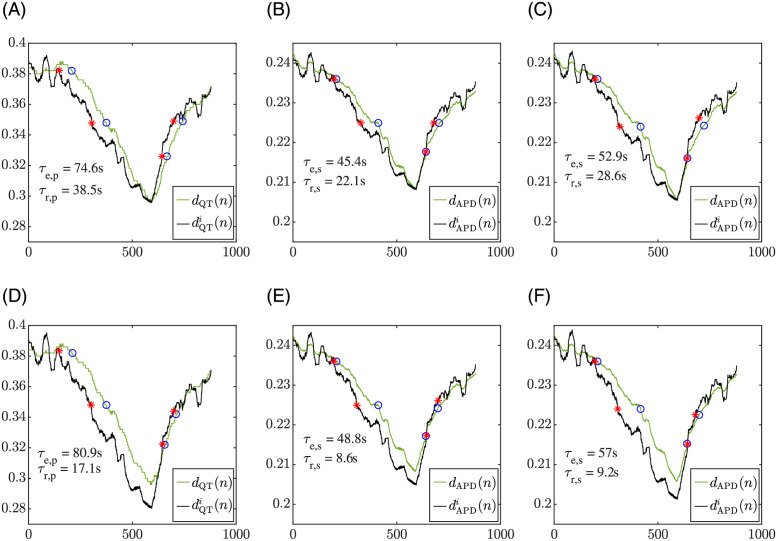
Left panels: QT adaptation delay between *d*_QT_(*n*) and $d_{\text{QT}}^{i}\left(n\right)$ during exercise and recovery for a patient in the study. Middle panels: APD adaptation delay between *d*_APD_(*n*) and $d_{\text{APD}}^{i}\left(n\right)$ during exercise and recovery in a simulated endocardial cell for constant *β*-adrenergic stimulation and the same HR as for the patient in the left. Right panels: APD adaptation delay between *d*_APD_(*n*) and $d_{\text{APD}}^{i}\left(n\right)$ during exercise and recovery in a simulated endocardial cell for the proposed time-varying *β*-adrenergic stimulation. Top panels show results using the U estimation strategy, while bottom panels use the M estimation strategy. The red points correspond to *n*_*e*, *o*_, *n*_*e*, *e*_, *n*_*r*, *o*_ and *n*_*r*, *e*_, calculated as described in section 2.6, which delimit the exercise and recovery ramps in $d_{\text{QT}}^{i}\left(n\right)$ or $d_{\text{APD}}^{i}\left(n\right)$. The blue points were identified in *d*_QT_(*n*) or *d*_APD_(*n*) as the nearest samples to the red points having QT or APD values within 2 ms of the corresponding red point value.

Fig 4 shows a comparison of the QT adaptation evaluated in a patient and the APD adaptation evaluated in an endocardial cell in response to the same HR changes, which correspond to the RR interval time series measured from the patient. The top left panel illustrates the *d*_QT_(*n*) and $d_{\text{QT}}^{i}\left(n\right)$ time series of a patient in response to the HR changes occurring during the stress test, with indication of the adaptation delay values during exercise and recovery. The top middle panel and the top right panel of the figure show the cellular *d*_APD_(*n*) and $d_{\text{APD}}^{i}\left(n\right)$ time series in response to the same HR changes of the patient with constant (Iso-c, middle panel) and time-varying (Iso-tv, right panel) Iso concentrations. Time delay values are indicated too. In all top panels, the $d_{\text{QT}}^{i}\left(n\right)$ or $d_{\text{APD}}^{i}\left(n\right)$ time series were calculated using the U strategy. The bottom panels of Fig 4 show analogous results using the M strategy. It can be noticed that *d*_APD_(*n*) series simulated for the constant and the time-varying Iso concentrations take the same values before *n*_1_ and after *n*_3_, since the Iso concentration coincides at those time segments. However, $d_{\text{APD}}^{i}\left(n\right)$ does not necessarily coincide for the constant and the time-varying Iso concentrations before *n*_1_ or after *n*_3_. This is so because this memoryless time series is computed from a regression model whose parameter values are estimated by fitting the [RR, APD] data in the windows *W*_1_, *W*_2_ and *W*_3_, with *W*_2_ containing the simulated APD values at the exercise peak, which differ for the constant and time-varying Iso concentrations.

Table 2 presents average values of the QT adaptation delays *τ*_*e*, *p*_ and *τ*_*r*, *p*_ across patients in each of the three CAD groups, ${\bar{\tau }}_{e,p}$ and ${\bar{\tau }}_{r,p}$, calculated using both the U and the M estimation strategies. Also, the average values of the APD adaptation delays calculated in a single endocardial cell are shown in the table for the constant and time-varying *β*-adrenergic stimulation patterns. As can be observed from the table, application of the proposed time-varying *β*-adrenergic stimulation pattern, Iso-tv, generally increased the mean value of the simulated APD delays, *τ*_*e*, *s*_ and *τ*_*r*, *s*_, making them closer to the QT adaptation delay measured in the patients as compared to the values obtained for constant *β*-adrenergic stimulation. This effect was better appreciated in the adaptation delay during the exercise phase, *τ*_*e*_, than during the recovery phase, *τ*_*r*_, and applied to both U and M estimation strategies, thus reinforcing the fact that Iso-tv better describes the repolarization adaptation to HR changes measured in the patients than Iso-c.

**Table 2:** Average values of the QT adaptation delays ${\bar{\tau }}_{e}$ and ${\bar{\tau }}_{r}$ (s) measured in the three CAD patient groups (third column), ${\bar{\tau }}_{e,p}$ and ${\bar{\tau }}_{r,p}$, and in a simulated endocardial cell, ${\bar{\tau }}_{e,s}$ and ${\bar{\tau }}_{r,s}$, with constant *β*-adrenergic stimulation (fourth column) and with the proposed time-varying *β*-adrenergic stimulation (fifth column).

Estimation Strategy	Patient Group	QT lag in patients		Simulated APD lag: Iso-c		Simulated APD lag: Iso-tv	
		${\bar{\tau }}_{e,p}$	${\bar{\tau }}_{r,p}$	${\bar{\tau }}_{e,s}$	${\bar{\tau }}_{r,s}$	${\bar{\tau }}_{e,s}$	${\bar{\tau }}_{r,s}$
U	low-CAD	51.1 ± 37.8	45.6 ± 36.2	33.6 ± 30.4	45.5 ± 20.6	42.5 ± 39.6	44.8 ± 24.7
	mild-CAD	31.9 ± 22.0	36.8 ± 12.2	−3.3 ± 8.3	32.7 ± 9.2	23.1 ± 18.5	36.1 ± 12.9
	high-CAD	56.2 ± 24.4	36.8 ± 16.4	44.1 ± 22.7	37.7 ± 28.1	54.9 ± 19.5	41.1 ± 35.8
M	low-CAD	69.0 ± 54.9	33.1 ± 34.6	43.2 ± 37.3	29.2 ± 31.3	68.8 ± 63.0	28.8 ± 32.8
	mild-CAD	50.9 ± 40.7	28.7 ± 11.0	−5.7 ± 14.2	32.5 ± 8.4	39.5 ± 31.4	30.1 ± 12.5
	high-CAD	71.1 ± 26.1	24.7 ± 17.2	60.2 ± 31.2	17.9 ± 30.3	73.2 ± 27.0	25.6 ± 41.9

A comparison of the time delays obtained for the four tested *β*-adrenergic stimulation is presented in S1 Table in S1 File. As can be observed, the individual, time-varying Iso-tv pattern led to adaptation delays that were in remarkably better agreement with those measured from the patients than the other two time-varying patterns, Iso-li and Iso-ab.

### 3.3 QT adaptation to HR changes in simulated pECGs

**Fig 5:**
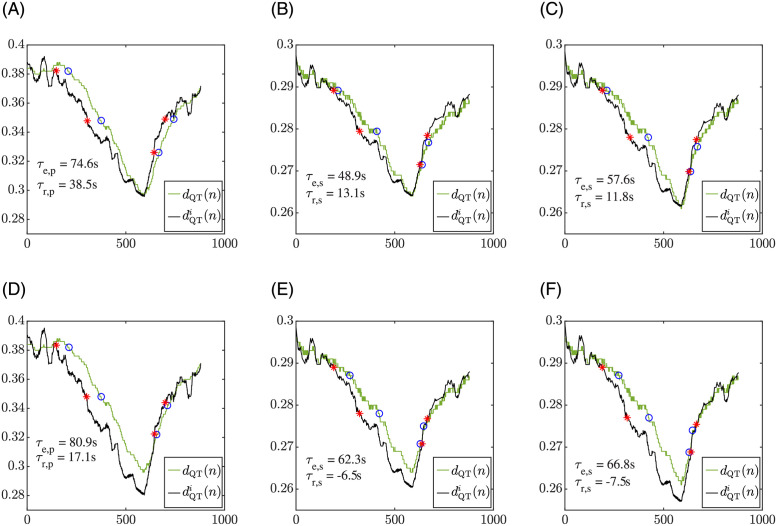
Left panels: QT adaptation delay between *d*_QT_(*n*) and $d_{\text{QT}}^{i}\left(n\right)$ during exercise and recovery for a patient in the study. Middle panels: QT adaptation delay between *d*_QT_(*n*) and $d_{\text{QT}}^{i}\left(n\right)$ during exercise and recovery in a simulated pECG for constant *β*-adrenergic stimulation and the same HR as for the patient in the left. Right panels: QT adaptation delay between *d*_QT_(*n*) and $d_{\text{QT}}^{i}\left(n\right)$ during exercise and recovery in a simulated pECG for the proposed time-varying *β*-adrenergic stimulation. Top panels show results using the U estimation strategy, while bottom panels use the M estimation strategy. The red points correspond to *n*_*e*, *o*_, *n*_*e*, *e*_, *n*_*r*, *o*_ and *n*_*r*, *e*_, calculated as described in section 2.6, which delimit the exercise and recovery ramps in $d_{\text{QT}}^{i}\left(n\right)$. The blue points were identified in *d*_QT_(*n*) as the nearest samples to the red points having QT values within 2 ms of the corresponding red point value.

Fig 5 shows a comparison of the QT adaptation in the same patient as in Fig 4 and the QT adaptation in a simulated pECG calculated from a tissue fiber in response to the patient’s HR changes during stress test. The left panels illustrate the QT adaptation to HR for the patient using the U (top) and M (bottom) estimation strategies. The middle and right panels of the figure show the QT adaptation to HR for the simulated tissue fiber under constant (Iso-c, middle) and time-varying (Iso-tv, right) Iso concentration patterns, using both the U (top) and M (bottom) estimation strategies. It can be noticed that, similarly to the observation for APD, *d*_QT_(*n*) series simulated for the constant and the time-varying Iso concentrations take the same values before *n*_1_ and after *n*_3_, but $d_{\text{QT}}^{i}\left(n\right)$ does not necessarily take the same values in those time segments.

**Fig 6:**
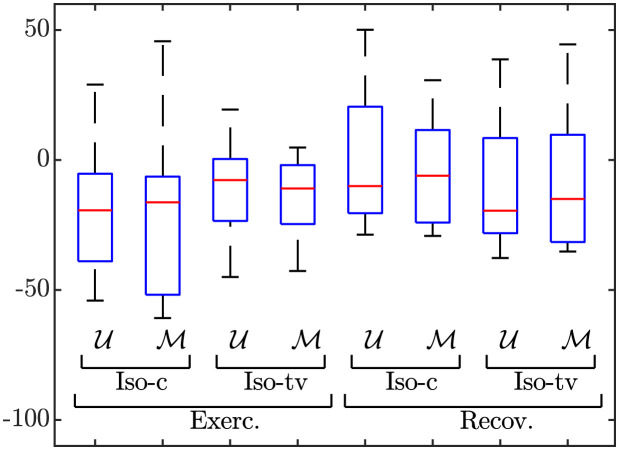
Distributions of the differences *Λ*_*τ*_ = *τ*_*φ*, *s*_ − *τ*_*φ*, *p*_, *φ* ∈ {*e*, *r*}, between the QT adaptation delays calculated in the simulations, *τ*_*φ*, *s*_, and the corresponding QT adaptation delays from the patients, *τ*_*φ*, *p*_, both under constant (Iso-c) and time-varying (Iso-tv) *β*-adrenegic stimulation, for exercise (four most-left boxplots) and recovery (four most-right boxplots).

Table 3 presents average values of the QT adaptation delays across patients in each of the three CAD groups during the exercise and recovery phases and across the simulated tissue fiber paced according to the different HR time series of the same set of patients, both under Iso-c and and Iso-tv *β*-adrenergic stimulation patterns. Results in the form of boxplots showing the differences between the QT adaptation delays in patients and in simulations are presented in Fig 6.

**Table 3:** Average values of the QT adaptation delays ${\bar{\tau }}_{e}$ and ${\bar{\tau }}_{r}$ measured in the three patient groups (third column), ${\bar{\tau }}_{e,p}$ and ${\bar{\tau }}_{r,p}$, and in a simulated endocardial cell, ${\bar{\tau }}_{e,s}$ and ${\bar{\tau }}_{r,s}$, with constant *β*-adrenergic stimulation (fourth column) and with the proposed time-varying *β*-adrenergic stimulation (fifth column).

Estimation Strategy	Patient Group	QT lag in patients		Simulated QT lag: Iso-c		Simulated QT lag: Iso-tv	
		${\bar{\tau }}_{e,p}$	${\bar{\tau }}_{r,p}$	${\bar{\tau }}_{e,s}$	${\bar{\tau }}_{r,s}$	${\bar{\tau }}_{,s}$	${\bar{\tau }}_{r,s}$
U	low-CAD	51.1 ± 37.8	45.6 ± 36.2	27.7 ± 27.6	54.2 ± 31.6	36.1 ± 26.0	47.7 ± 30.5
	mild-CAD	31.9 ± 22.0	36.8 ± 12.2	5.4 ± 19.3	36.9 ± 7.1	16.6 ± 37.8	25.8 ± 8.3
	high-CAD	56.2 ± 24.4	36.8 ± 16.4	47.9 ± 22.5	26.2 ± 18.5	48.6 ± 24.9	18.8 ± 18.6
M	low-CAD	69.0 ± 54.9	33.1 ± 34.6	35.7 ± 33.2	34.4 ± 20.8	50.5 ± 37.4	23.1 ± 24.2
	mild-CAD	50.9 ± 40.7	28.7 ± 11.0	25.3 ± 29.6	28.5 ± 10.1	23.4 ± 61.5	28.8 ± 35.4
	high-CAD	71.1 ± 26.1	24.7 ± 17.2	67.4 ± 33.9	9.2 ± 25.9	66.7 ± 26.5	7.2 ± 27.6

**Fig 7:**
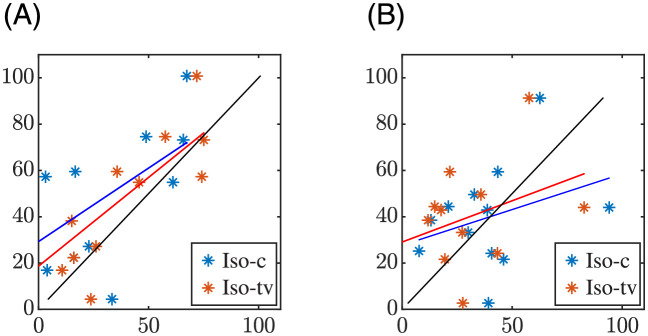
Relationship between the QT adaptation delays calculated using the U estimation strategy in patients (vertical axis, *τ*_*φ*, *p*_, *φ* ∈ {*e*, *r*}) and in simulations (horizontal axis, *τ*_*φ*, *s*_) for a constant (blue) or time-varying Iso level (red). Fitted lines are shown in the corresponding colors, while black lines show the diagonal. The left panel corresponds to the exercise phase and the right panel to the recovery phase.

**Fig 8:**
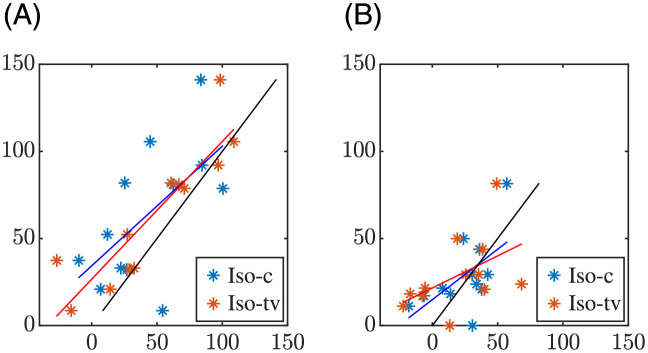
Relationship between the QT adaptation delays calculated using the M estimation strategy in patients (vertical axis, *τ*_*φ*, *p*_, *φ* ∈ {*e*, *r*}) and in simulations (horizontal axis, *τ*_*φ*, *s*_) for a constant (blue) or time-varying Iso level (red). Fitted lines are shown in the corresponding colors. The black lines show the diagonal.

Fig 7 shows the relationship between the QT adaptation delays measured in patients and those obtained in simulations for constant (Iso-c) and time-varying (Iso-tv) *β*-adrenergic stimulation using the U estimation strategy. Analogous results for the M estimation strategy are depicted in Fig 8.

## 4. Discussion

We used computational modeling and simulation to characterize the role of *β*-adrenergic stimulation in contributing to QT interval adaptation after HR changes during a stress test. We first quantified the QT interval adaptation delay in a set of CAD patients with mild-to-severe coronary occlusion, separately for the exercise and recovery phases of the stress test. We next simulated the electrical activity of single ventricular cells and a ventricular tissue fiber paced at each of the HR time series measured from the patients. We evaluated the APD adaptation delay from the cells and the QT interval adaptation delay from a pECG generated by the tissue fiber. We found that, when the cells and tissue were subjected to an individual, time-varying *β*-adrenergic stimulation pattern with higher stimulation levels around the stress peak, the simulated adaptation delays were in better agreement with the adaptation delays measured from the patients than when subjected to a constant, baseline *β*-adrenergic stimulation level, particularly during the exercise phase of the test. Other *β*-adrenergic stimulation patterns defined by a constant, high Iso concentration along exercise or by a linearly increasing Iso concentration all along the exercise phase rendered adaptation delays that were not in such good agreement with those from the patients. These results highlight the role of individual, time-varying *β*-adrenergic stimulation patterns in explaining the repolarization adaptation to HR changes.

### 4.1 QT rate adaptation in CAD patients

We started by analyzing a reduced set of stress test recordings from CAD patients. We quantified the QT interval adaptation delay in exercise and recovery based on the difference between the measured QT time series and an estimated memoryless QT time series obtained by inputting the RR time series to a linear or hyperbolic regression model. The fitting of these regression models was performed in windows where the RR interval was approximately stationary, with the M strategy being further developed than the U strategy to compensate for the lack of stationarity in the proximity of the stress peak. Although the inter-patient variability in the QT adaptation lags was very high, which is in line with prior works on rate adaptation of repolarization [14, 17, 20–22, 57], we were able to observe that the QT interval delays calculated using the M strategy were longer during exercise than during recovery, with the mean adaptation time during exercise being around twice the mean adaptation time during recovery. Only when closely approaching the stress peak, the QT adaptation lag after HR became progressively diminished, as noted by a reduction in the difference between the measured and the estimated memoryless QT time series.

### 4.2 Role of *β*-adrenergic stimulation in APD and QT rate adaptation

We next used *in silico* cell and tissue models coupling descriptions of human ventricular electrophysiology and *β*-adrenergic signaling. We considered single cells and a transmural tissue fiber composed of cells from the subendocardium, midmyocardium and subepicardium and we paced both the single cells and the tissue according to the HR time series of each patient of the study. We calculated the cellular APD and the QT interval of a pECG generated by the tissue fiber in response to the HR changes of the patients both for *β*-adrenergic stimulation fixed at a constant, baseline level and for *β*-adrenergic stimulation varying along time. We concluded that a *β*-adrenergic stimulation pattern with high Iso levels around the stress peak showed the best results in terms of replicating the repolarization adaptation measured from the patients. The APD and QT interval delays were, in general, lower in the simulated cells and pECGs than in the patients’ ECGs. Importantly, when comparing the results obtained after application of a time-varying *β*-adrenergic stimulation pattern with respect to the ones obtained for constant *β*-adrenergic stimulation, we found that the APD and QT adaptation delays during exercise, as calculated with the M strategy, were closer to the QT delays measured from the patients. This is illustrated for the QT interval in Fig 6 (left-most boxplots) and in Fig 8 (left panel), which show the distributions of the delay differences and the delays’ correlation, respectively, between simulated pECGs and patients’ ECGs. During recovery, the differences in the APD and QT adaptation delays between the constant and time-varying *β*-adrenergic stimulation patterns were not as evident as during exercise when calculated using the M strategy. Our results on the relevant role of *β*-adrenergic stimulation in contributing to rate adaptation of repolarization are in agreement with previous studies in the literature [22, 28, 36, 58, 59]. Also, the fact that the QT adaptation delay became progressively reduced when approaching the peak of the stress test is in line with *in silico* cell studies reporting that the higher the pre-stimulation level of *β*-adrenoceptors, the shorter the APD adaptation time [36].

### 4.3 Differential role of *β*-adrenergic stimulation as a function of the extent of disease

Separately analyzing the results for the three CAD groups, with mild-to-severe coronary occlusion, we found that the role of the *β*-adrenergic stimulation seemed to be more relevant in the low-CAD group than in the mild-CAD and high-CAD groups, although further analysis on larger number of patients is required. This observation was confirmed by the results in Table 3, which show that the differences between the simulated QT delays under constant and time-varying *β*-adrenergic stimulation are large in the low-CAD group but only minor in the other two groups. While application of the time-varying *β*-adrenergic stimulation led to simulated QT delays closer to the patients’ QT delays during the exercise phase, the opposite held during the recovery phase. Further investigations could account for remodeling in ventricular electrophysiology and/or *β*-adrenergic signaling as a function of the extent of the disease to uncover the differential role of *β*-adrenergic stimulation in the three analyzed groups [34, 60–62]. Of relevance, the use of cell and tissue models of diseased ventricles could lead to larger QT adaptation delays in the simulations, as previous studies have reported protracted QT adaptation in diseases associated with impairment of the sodium-potassium pump activity such as heart failure, ischemic heart disease or hypertension [46, 63–66]. This would be expected to render simulated QT delays in better accordance with the QT delays quantified in our patient population (dots in Figs 7 and 8 closer to the diagonal).

### 4.4 QT rate adaptation can be explained by cellular rate adaptation dynamics

Our simulation results showed that the rate adaptation dynamics of the QT interval and the cellular APD were similar, in agreement with previous studies investigating repolarization adaptation to other types of HR changes, like abrupt HR changes, at different ventricular scales [46]. Here, we observed that simulated APD delays in single subendocardial cells were even closer to the QT delays measured from the patients than the simulated QT delays. This could be partially explained by the fact that the simulated tissue fiber was composed of subendocardial, midmyocardial and subepicardial cells, with quantitative differences in the APD adaptation delay of the three cell types.

### 4.5 Study limitations and future work

In our modeling and simulation study, we used a transmural fiber with a unique transmural composition and with no specific CAD characterization. Future studies could assess the extent to which the transmural composition and the degree of CAD-induced remodeling in ventricular electrophysiology and *β*-adrenergic signaling impact the simulated QT delay. A procedure could be developed to define the tissue models so that they more closely reproduce the clinically measured QT delays in different patient groups. These type of studies and others using more personalized *in silico* models, possibly based on QT adaptation delays quantified in larger study populations, could help to confirm the outcomes of our present research. Additionally, our modeling presents limitations in terms of explaining the QT adaptation delays during recovery from exercise. In the future, different time-varying patterns of *β*-adrenergic stimulation, particularly during the recovery phase of the stress test, could be identified to more accurately reproduce the adaptation times measured from the patients and establish the underlying mechanisms. Finally, non-corrected QT interval values were used here to estimate the adaptation time lag in stress test recordings and assess the underlying mechanisms. Future works could take the estimated values of the time lag and the parameters of the optimally fitted regression model and use them to correct the QT interval for the effects of HR, following an approach similar to that proposed in previous studies (Pueyo et al. 2004, Smetana et al. 2004).

### 4.6 Novelty and relevance of the study

Previous investigations evaluated the effects of additional covariates apart from HR, like sympathetic activation, on the QT interval during exercise, by using HR correction formulas. However, the results from these investigations were not conclusive [67, 68]. In other studies, autonomic blockades were used to assess QT hysteresis during exercise and recovery, providing evidence on the important role of autonomic nervous system effects on the QT-to-RR relationship [59]. Nevertheless, subsequent reports pointed out that the conclusions of those studies might be influenced by factors like the speed of HR change, which is also subjected to autonomic influence [69]. Here, we aimed to shed light on the role of *β*-adrenergic stimulation on the APD and QT adaptation lag after HR changes separately during exercise and recovery. Importantly, we sought to propose a possible description of the *β*-adrenergic stimulation pattern that could explain the repolarization adaptation after HR in stress test recordings. To the best of our knowledge, there is not any study proposing individualized, time-varying patterns of *β*-adrenergic stimulation to describe the QT lag after RR during exercise and recovery in CAD patients.

The relevance of our results can be understood on the basis of the increased risk of sudden cardiac death during or just after exercise. Among the possible sources for this increased risk, the changes in cardiac autonomic modulation that accompany exercise have been suggested to be important contributors [70]. Autonomic changes greatly affect ventricular repolarization, in general, and the QT interval, in particular, due to the autonomic action on the ventricular myocardium, on top of influencing the HR as a result of the autonomic control of the sinoatrial node activity. Since such changes may be associated with the generation of ventricular arrhythmias, the investigation of the relationship between the QT interval and the HR in stress test recordings, with specific evaluation of the rate adaptation of the QT interval and its underpinnings, becomes of major importance. Specifically, identification of the individual, time-varying *β*-adrenergic stimulation pattern underlying QT rate adaptation during exercise and right after could help to understand why some patients are at higher arrhythmic risk than others and could represent a basis for future studies aimed at designing approaches to reduce the risk.

## 5. Conclusions

We quantified the delay in the QT interval adaptation to HR changes in a set of CAD patients undergoing a stress test. By *in silico* modeling and simulation of cell and tissue ventricular electrophysiology and *β*-adrenergic signaling, we showed that *β*-adrenergic stimulation modulates QT interval adaptation to HR in stress tests. Specifically, a time-varying pattern of *β*-adrenergic stimulation with higher stimulation levels around the stress peak helped to better reproduce the QT rate adaptation delays quantified from CAD patients than a constant level of *β*-adrenergic stimulation, particularly during the exercise phase of the test. The role of *β*-adrenergic stimulation in the adaptation of the QT interval seemed to be more relevant in patients with low degree of coronary occlusion than in those with mild or high degree and was well described by the cellular dynamics of APD rate adaptation.

## Supporting information

S1 File Tested *β*-adrenergic stimulation patterns. Simulated APD responses to HR changes for the four analyzed patterns of *β*-adrenergic stimulation.
(PDF)
