# Supplementary material for: The role of β-adrenergic stimulation in QT interval adaptation to heart rate during stress test
Source: PLoS One. 2023 Jan 26;18(1):e0280901. doi: 10.1371/journal.pone.0280901 (PMC9879473; doi:10.1371/journal.pone.0280901)
Supplement: S1 File — Simulated APD responses to HR changes for the four analyzed patterns of β-adrenergic stimulation. (PDF) [file pone.0280901.s001.pdf]

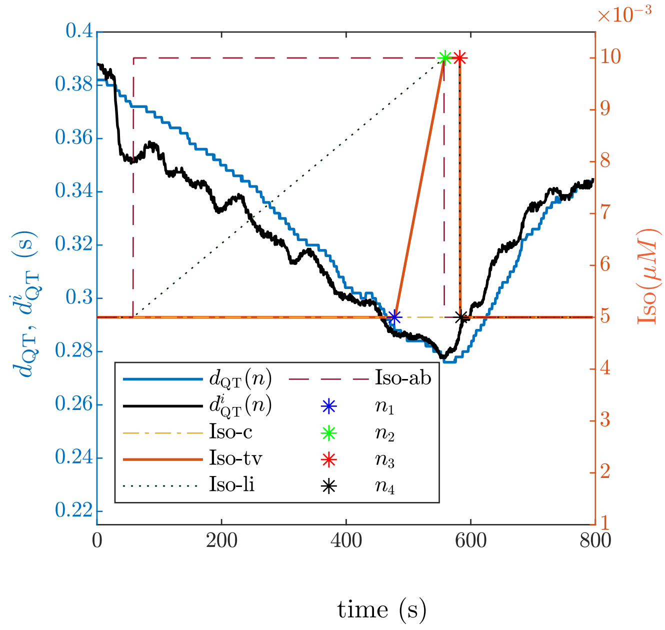

Figure 1: Simulated patterns of Iso concentrations during exercise and recovery. The constant pattern (Iso-c) was defined by a constant, baseline level of Iso equal to 0.005 μM. The time-varying pattern (Iso-tv) was defined by time points n_1, n_2, n_3 and n_4 determined from the $d^{i}_{\mathrm{QT}}(n)$ and $d_{\mathrm{QT}}(n)$ time series of an analyzed ECG recording from a CAD patient. The linearly increasing pattern (Iso-li) was defined as the Iso-tv pattern but with an Iso concentration varying linearly along exercise from the baseline level of 0.005 μM to 0.01 μM. The abruptly changing pattern (Iso-ab) was defined as the Iso-tv pattern but with an abrupt change in Iso at the beginning of exercise from 0.005 μM to 0.01 μM, which remained at this value until the exercise peak.

Table 1 presents average values of the QT adaptation delays $\tau_{e,p}$ and $\tau_{r,p}$ across patients in each of the three CAD groups, $\overline{\tau}_{e,p}$ and $\overline{\tau}_{r,p}$, calculated for the constant and the three different time-varying β-adrenergic stimulation patterns using both the $\mathcal{U}$ and the $\mathcal{M}$ estimation strategies. Also, the average values of the APD adaptation delays calculated in a single endocardial cell are shown in the table for all the patterns. As can be observed from the table, application of the individualized, time-varying β-adrenergic stimulation pattern Iso-tv generally increased the mean value of the simulated APD delays, $\tau_{e,s}$ and $\tau_{r,s}$, making them closer to the QT adaptation delay measured in the patients as compared to the values obtained for the rest of β-adrenergic stimulation patterns. This effect was better appreciated in the adaptation delay during the exercise phase, τ_e, than during the recovery phase, τ_r, and applied to both $\mathcal{U}$ and $\mathcal{M}$ estimation strategies, thus reinforcing the fact that the time-varying Iso-tv pattern better describes the repolarization adaptation to HR changes measured in the patients than the constant Iso-c pattern and any of the two other patterns.

Table 1: Average values of the QT adaptation delays $\overline{\tau}_e$ and $\overline{\tau}_r$ (s) measured in the three CAD patient groups (third column), $\overline{\tau}_{e,p}$ and $\overline{\tau}_{r,p}$, and in a simulated endocardial cell, $\overline{\tau}_{e,s}$ and $\overline{\tau}_{r,s}$, for the four analyzed β-adrenergic stimulation patterns.

Estimation Strategy		Iso pattern	τ		low-CAD		mild-CAD		high-CAD	
					τ_e	τ_r	τ_e	τ_r	τ_e	τ_r
$\mathcal{U}$	**QT lag**	Patients	$\overline{\tau}_{e,p}$	$\overline{\tau}_{r,p}$	51.1 ± 37.8	45.6 ± 36.2	31.9 ± 22.0	36.8 ± 12.2	56.3 ± 24.3	36.8 ± 16.4
	**Simulated APD lag**	**Iso-ct**	$\overline{\tau}_{e,s}$	$\overline{\tau}_{r,s}$	33.6 ± 30.4	45.5 ± 20.6	-3.3 ± 8.3	32.7 ± 9.2	44.1 ± 22.7	37.8 ± 28.1
		**Iso-tv**			42.5 ± 39.6	44.8 ± 24.7	23.1 ± 18.5	36.1 ± 12.9	54.9 ± 19.5	41.1 ± 35.8
		**Iso-li**			12.8 ± 36.0	37.3 ± 18.2	-5.7 ± 14.1	33.5 ± 14.8	48.7 ± 27.4	36.4 ± 39.1
		**Iso-ab**			-31.0 ± 26.4	31.7 ± 20.5	-51.6 ± 15.9	27.7 ± 18.6	-2.8 ± 28.7	21.9 ± 24.7
$\mathcal{M}$	**QT lag**	Patients	$\overline{\tau}_{e,p}$	$\overline{\tau}_{r,p}$	69.0 ± 54.9	33.1 ± 34.6	50.9 ± 40.7	28.7 ± 11.0	71.1 ± 26.1	24.7 ± 17.2
	**Simulated APD lag:**	**Iso-ct**	$\overline{\tau}_{e,s}$	$\overline{\tau}_{r,s}$	43.2 ± 37.3	29.2 ± 31.3	-5.7 ± 14.2	32.5 ± 8.4	60.2 ± 31.2	17.9 ± 30.3
		**Iso-tv**			68.8 ± 63.0	28.8 ± 32.8	39.5 ± 31.4	30.1 ± 12.5	73.2 ± 27.0	25.6 ± 41.9
		**Iso-li**			19.6 ± 50.2	33.8 ± 33.3	-4.8 ± 25.5	36.0 ± 10.1	69.0 ± 42.8	19.3 ± 33.7
		**Iso-ab**			-40.0 ± 33.0	42.4 ± 27.3	-79.2 ± 18.7	39.9 ± 7.7	-1.65 ± 39.3	28.4 ± 37.6
